# Impact of chronic obstructive pulmonary disease on linear and nonlinear dynamics of heart rate variability in patients with heart failure

**DOI:** 10.1590/1414-431X202010084

**Published:** 2020-11-27

**Authors:** C.L. Goulart, F.R. Caruso, G.P.T. Arêas, P.B. dos Santos, P.F. Camargo, L.C.S. de Carvalho, M.G. Roscani, R.G. Mendes, A. Borghi-Silva

**Affiliations:** 1 Laboratório de Fisioterapia Cardiopulmonar, Departamento de Fisioterapia, Universidade Federal de São Carlos, São Carlos, SP, Brasil; 2 Laboratório de Fisiologia Humana, Departamento de Fisiologia, Universidade Federal do Amazonas, Manaus, AM, Brasil; 3 Centro de Ciência e Tecnologia em Energia e Sustentabilidade, Universidade Federal do Reconcavo da Bahia, Feira de Santana, BA, Brasil; 4 Departamento de Medicina, Universidade Federal de São Carlos, São Carlos, SP, Brasil

**Keywords:** Exercise test, Cardiovascular disease, Heart failure, COPD, Heart rate

## Abstract

The objective of this study was to investigate the impact of chronic obstructive pulmonary disease (COPD)-heart failure (HF) coexistence on linear and nonlinear dynamics of heart rate variability (HRV). Forty-one patients (14 with COPD-HF and 27 HF) were enrolled and underwent pulmonary function and echocardiography evaluation to confirm the clinical diagnosis. Heart rate (HR) and R-R intervals (iRR) were collected during active postural maneuver (APM) [supine (10 min) to orthostasis (10 min)], respiratory sinus arrhythmia maneuver (RSA-M) (4 min), and analysis of frequency domain, time domain, and nonlinear HRV. We found expected autonomic response during orthostatic changes with reduction of mean iRR, root mean square of successive differences between heart beats (RMSSD), RR tri index, and high-frequency [HF (nu)] and an increased mean HR, low-frequency [LF (nu)], and LF/HF (nu) compared with supine only in HF patients (P<0.05). Patients with COPD-HF coexistence did not respond to postural change. In addition, in the orthostatic position, higher HF nu and lower LF nu and LF/HF (nu) were observed in COPD-HF compared with HF patients. HF patients showed an opposite response during RSA-M, with increased sympathetic modulation (LF nu) and reduced parasympathetic modulation (HF nu) (P<0.05) compared with COPD-HF patients. COPD-HF directly influenced cardiac autonomic modulation during active postural change and controlled breathing, demonstrating an autonomic imbalance during sympathetic and parasympathetic maneuvers compared with isolated HF.

## Introduction

The coexistence of chronic obstructive pulmonary disease (COPD) in patients with heart failure (HF) leads to severe impairments in functional capacity (1) and quality of life, and both diseases have important systemic components that affect autonomic adjustments and functioning of several systems, such as cardiovascular control at rest and during exercise (2,3). The cardiovascular system and the mechanisms that regulate autonomic adjustments can be investigated by analyzing heart rate variability (HRV), which represents a powerful tool for research capable of identifying increased mortality risk and poor prognosis (4).

HF patients present sympathetic-vagal imbalance of the sinus node, with a predominance of sympathetic tone (5). In addition, limitations in cardiac function compromise the transport of nutrients and metabolic products from the organic system and cause sympathetic hyperactivity and consequent decrease in vagal tone (6). These patients may present with chronic hypoxemia of the peripheral tissue, which is capable of modifying the control by central and peripheral chemoreceptors (7).

COPD has a major impact on systemic manifestations such as attenuated HRV responses, increased sympathetic activity, and resting heart rate (HR). These responses may be related to marked parasympathetic airway hyperactivity, bronchoconstriction and vasoconstriction, hypoxemia, hypercapnia, and systemic inflammation (8–10). Zangrando et al. (11) found that autonomic modulation during active postural maneuver (APM) was impaired with parasympathetic modulation predominating. The APM is a powerful stimulus to increase sympathetic modulation, and when its response is absent, it may indicate vagal resumption failure with consequent sympathetic hyperactivity, which may directly influence exercise response (10).

These autonomic imbalances may have a negative impact on static postural adjustments and during respiratory maneuvers in these patients; however, there is no study evaluating APM and respiratory sinus arrhythmia maneuver (RSA-M) in coexisting COPD-HF patients. Therefore, our aim was to evaluate the impact of coexisting COPD-HF on linear and nonlinear dynamics of HRV by both stimulus APM and RSA-M. We hypothesized an impaired autonomic response in COPD-HF patients compared with HF patients considering a higher resting sympathetic status.

## Material and Methods

### Study design

This cross-sectional study was carried out according to the recommendations of the STROBE statement. The study followed the Declaration of Helsinki and it was approved by the Universidade Federal de São Carlos (protocol number: 91088318.7.1001.5504). All volunteers signed a written informed consent statement prior to participation.

### Subjects

#### Inclusion Criteria

Patients with a clinical diagnosis of COPD and evidenced by pulmonary function tests [FEV_1_/forced vital capacity (FVC) ratio of 0.7; FEV_1_ 60% of predicted] (12) and a clinical diagnosis of HF in patients with a left ventricle ejection fraction-LVEF <50% (13), >50 years of age, and HF class according to New York Heart Association Functional Classification (NYHA) (14) were included in the study.

#### Exclusion Criteria

All patients that presented previous COPD or HF exacerbations (clinical care with medication change, need for antibiotics, addition of inotropes, or need for hospitalization), patients that presented concomitant musculoskeletal disorders or neurological conditions affecting the locomotor system that impaired the postural position protocol, cognitive impairment, or comprehension deficiencies assessed by the Mini Mental State test, clinical diagnoses of lung cancer, heavy alcohol drinkers, electrocardiographic abnormalities (e.g., atrial fibrillation and left bundle branch block), unstable angina, and uncontrolled metabolic and cardiac diseases were excluded.

### Protocol

All patients underwent an echocardiogram administered by a cardiologist, a pulmonary function exam performed by a pulmonologist, and a clinical assessment. Every patient completed the comprehensive evaluation process in three days: 1) clinical evaluation by a physician and a physical therapist; 2) lung function test and Doppler echocardiography; and 3) R-R intervals (iRR) and HR assessment during supine and orthostatic position and RSA-M.

### Measurements

#### Doppler echocardiography

Initially for the clinical and diagnostic stratification, the COPD-HF patients underwent a 2D-echocardiogram using an iE33 system (Philips, USA) with a 2-5 MHz matrix transducer and tissue Doppler imaging software. The same physician assessed all patients and they were instructed to lie on the left side of their body. Quantifications of the cardiac chambers were performed according to the American Society of Echocardiography (15).

#### Pulmonary function

Pulmonary function was obtained using a digital spirometer (Breeze^®^, Medgraphics, MGC Diagnostics Corporation, USA) that provided measurements of the forced expiratory volume in the 1st second (FEV_1_) and the forced vital capacity (FVC), enabling the calculation of the FEV_1_/FVC ratio. Spirometry was performed according to the recommendations of the American Thoracic Society/European Respiratory Society guidelines (16). The classification of severity of airflow limitation in COPD was performed according to the Global Initiative for Chronic Obstructive Lung Disease (GOLD) recommendations and patients were classified as moderate (GOLD II), severe (GOLD III), or very severe (GOLD IV) (17).

### Heart rate and iRR recordings

Patients were evaluated in a laboratory at a temperature of 22°C and relative humidity between 50 and 60%. They were instructed to avoid stimulants and alcoholic drinks and not to perform exhausting physical exercise the day before the test; they were also instructed not to smoke or use bronchodilators for 6 h before the test. On the day of the test, guidelines were given to patients to avoid sleeping and to not speak or move their arms and legs during data collection. It was advised, however, that if the patient manifested any discomfort or symptom of dizziness, tiredness, or fatigue, they could request to interrupt the measurement at any time.

HR and iRR were recorded using PowerLab^®^ electrocardiographs (ADIntruments, Australia) used in MC5 lead, captured and stored by LabChart^®^ v. 8.0 software (ADIntruments), with a sampling rate of 500 Hz and 1 ms time resolution. All artifacts were reviewed by visual inspection on the computer display. Only segments with >90% pure sinus beats were included in the final analysis (18). Recorded signals contained at least 256 points for APM analysis (18) and the data were then transferred to Kubios HRV^®^ software (version 2.2, Finland).

#### Active postural maneuver

After a period of rest in the supine position to prepare the patient for the experimental conditions and placement of the ECG electrodes (approximately 10 min), HR and iRR were recorded for 10 min. After this rest period, the subjects were instructed to remain standing, without moving or speaking for another 10 min (18), and finally, spontaneous breathing (SB) in the sedestation position was analyzed. During APM, we analyzed the 256 most stable points.

#### Respiratory sinus arrhythmia maneuver

**Figure 1:**
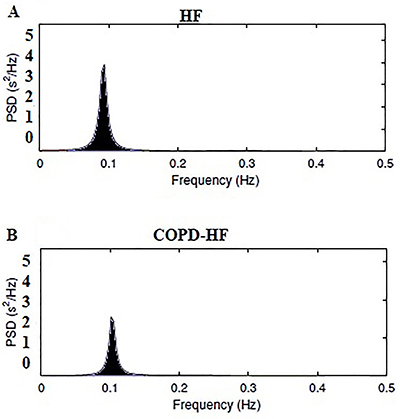
Decomposition of the spectrum into single spectral components in respiratory sinus arrhythmia maneuver (RSA-M). **A**, Heart failure (HF) patient (2' 30'' of RSA-M). **B**, Chronic obstructive pulmonary disease-heart failure (COPD-HF) patient (2' 40'' of RSA-M). PSD: total spectral power.

Subjects were instructed to perform a series of deep and slow inspirations and expirations, with a pulmonary volume that varied from the total lung capacity (maximal inspiration) to the residual volume (maximal expiration) (5). Each respiratory cycle was performed in 10 s, with a 5-s inspiration and a 5-s expiration, resulting in six respiratory cycles per minute. The results of RSA-M were compared with the spontaneous breathing (10 min). Analyses of time, frequency, and non-linear domains were also performed during RSA-M and the most stable breathing cycles of the maneuver were analyzed (2-min), which were performed with 5-6 breaths per minute. The spectral analysis confirmed that the volunteers maintained a correct respiratory rate, which corresponds to a peak frequency of spectral density between 0.08 and 0.1 Hz (Figure 1) (19).

### Cardiac autonomic modulation analysis - HRV indices

Frequency domain, time domain, and nonlinear analysis were performed on signals recorded during the RSA-M. Time domain analysis provided mean iRR, mean HR, root mean square of successive differences between iRR (RMSSD), and iRR tri-index (9). Spectral analysis provided the HRV signal power in the low-frequency (LF) band, which is described per 0.04 to 0.15 Hz and in the high-frequency [HF (nu)] band, which is described per 0.15 to 0.4 Hz, and the LF/HF ratio reported in normalized units (nu) (8). Nonlinear analysis provided the plot de Poincaré (SD1 and SD2), alpha 1 (α1) (short-term fluctuation slope of the detrended fluctuation analysis) and alpha 2 (α2) (long-term fluctuation slope of the detrended fluctuation analysis), approximate entropy (ApEn), and the sample entropy (SampEn) indices (20).

### Statistical analysis

Data were analyzed using Graphpad Prism 7.0 (GraphPad Software, USA). The Shapiro-Wilk test was used to verify the data distribution. Descriptive data are reported as means±SD, frequency, and 95%CI (minimum and maximum values). The parametric Student's *t*-test was used for normally distributed data.

The difference regarding the Δ was calculated considering the supine position - orthostatic position (10 min), and the unpaired Student's *t*-test was applied for between-group comparisons. The statistical significance level was set at P<0.05.

## Results

**Figure 2:**
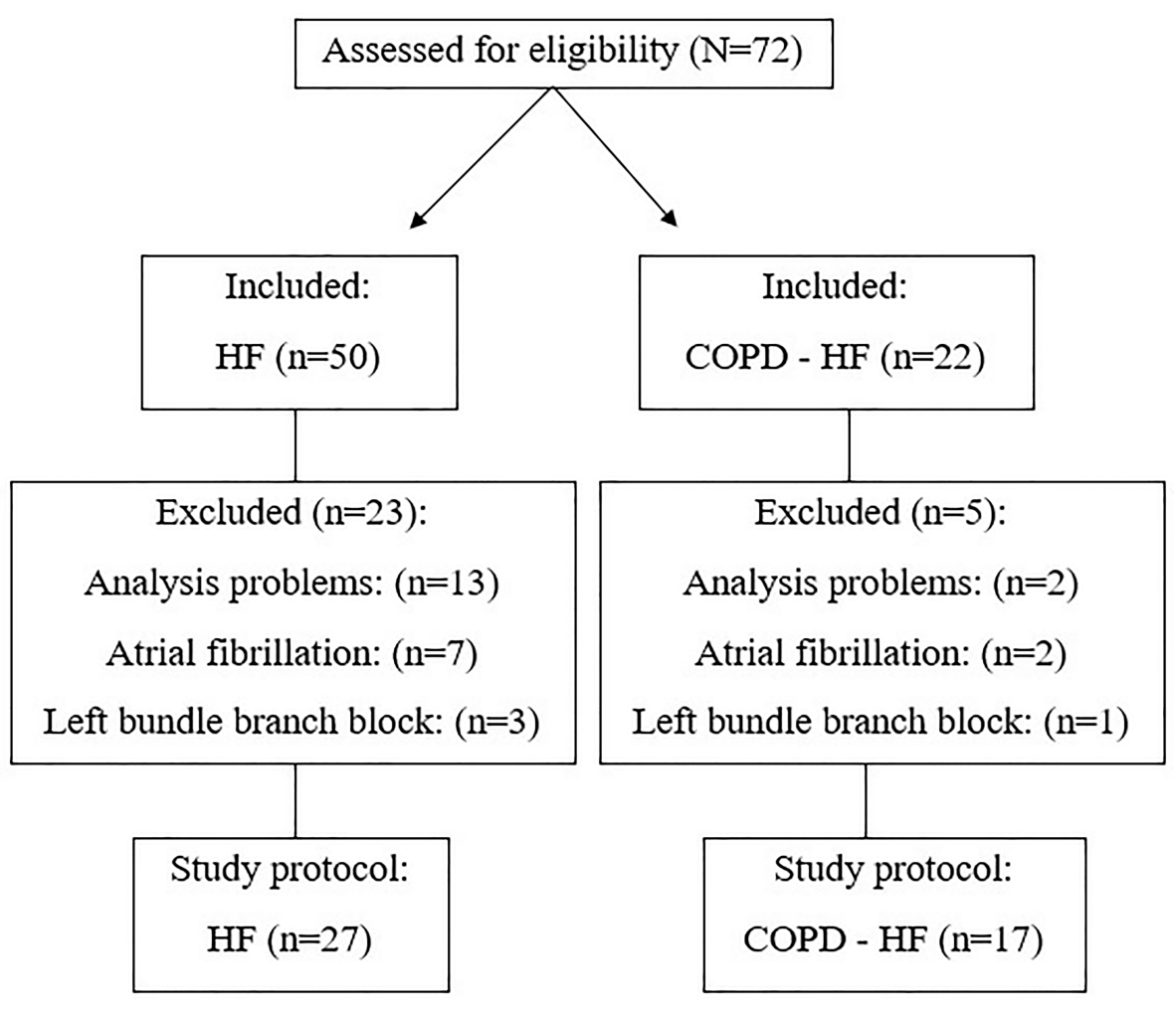
Flowchart of the study. HF: heart failure; COPD: chronic obstructive pulmonary disease.

Initially, we evaluated 37 HF patients, of which 10 were excluded due to electrocardiographic atrial abnormalities [fibrillation (n=7) and left bundle branch block (n=3)] and 17 COPD-HF patients, of which 3 were excluded due to electrocardiographic atrial abnormalities [atrial fibrillation (n=2) and left bundle branch block (n=1)] (Figure 2).

Table 1 shows the clinical, echocardiogram, and spirometry characteristics in HF and COPD-HF patients. As expected, COPD-HF patients had worse pulmonary function compared to HF patients; however, both groups were similar regarding the other characteristics.

**Table 1:** Clinical, echocardiogram, and spirometry's characteristics in heart failure (HF) patients and chronic obstructive pulmonary disease-heart failure (COPD-HF) patients.

Variables	HF (n=26)	COPD-HF (n=14)	P value
Male, n (%)	20 (76)	14 (100)	0.06
Age (years)	59±6	69±7	0.06
BMI (kg/m^2^)	30±6	28±8	0.28
LVEF (%)	41±5	40±6	0.70
NYHA, n (%)			0.08
I	14 (54)	7 (50)	-
II	10 (38)	3 (21)	-
III	2 (8)	4 (29)	-
Medications, n (%)			
β-blocker	26 (100)	14 (100)	-
Β_2_-agonists	-	14 (100)	-
Diuretics	16 (61)	8 (57)	0.07
Statins	5 (19)	3 (21)	0.02
ACE inhibitor	14 (54)	6 (43)	0.44
Platelet aggregation inhibitor	18 (69)	8 (57)	0.58
Digoxin	4 (15)	3 (21)	0.23
Inhaled corticosteroid	-	7 (50)	-
Pulmonary Function			
FEV_1_ (L)	2.7±0.9	1.8±0.7	0.004
% predicted	89±18	59±20	0.001
FVC (L)	3.6±0.9	3.2±0.8	0.29
% predicted	87±14	80±25	0.34
FEV_1_/FVC (L)	0.78±0	0.56±0.1	0.001
GOLD, n (%)			NA
I	-	5 (35)	-
II	-	4 (30)	-
III	-	5 (35)	-

Data are reported as mean±SD or n (%). Student's *t*-test was used to compare groups. LVEF: left ventricular ejection fraction; NYHA: New York Heart Association; BMI: body mass index; FVC: forced vital capacity; FEV_1_: forced expiratory volume in the 1st second; GOLD: Global Initiative for Chronic Lung Disease score.

### HRV indices in APM

We found expected autonomic response during orthostasis with reduction of mean iRR, RMSSD, RR tri-index, and HF nu and an increase in mean HR, LF nu, and LF/HF nu compared with supine (P<0.05) only in HF patients. However, time and frequency indices were not able to demonstrate responses to APM in patients with COPD-HF overlap. Only the sample entropy, non-linear index, showed a reduction in its values after the APM, demonstrating a reduction in the HR complexity from the supine to the orthostatic position (P<0.05). Patients with COPD-HF coexistence showed higher HF nu, lower LF nu, and LF/HF nu compared with HF patients in the orthostatic position (Table 2).

**Table 2:** Heart rate variability indices during active postural maneuver in heart failure (HF) patients and chronic obstructive pulmonary disease-heart failure (COPD-HF) patients.

	HF (n=27)		COPD-HF (n=14)	
	Supine	Orthostasis	Supine	Orthostasis
Time domain				
Mean HR	67 (63 to 72)	77 (71 to 82)*	75 (68 to 82)	82 (73 to 91)
Mean iRR	907 (850 to 963)	803 (746 to 859)*	818 (744 to 893)	753 (680 to 827)
RMSSD	33 (19 to 47)	22 (10 to 34)*	27 (3 to 51)	28 (8 to 48)
RR tri-index	6 (4 to 8)	4 (3 to 5)*	4 (2 to 7)	5 (3 to 7)
Frequency domain				
LF (nu)	49 (41 to 58)	62 (55 to 70)*	52 (35 to 68)	47 (36 to 59)^+^
HF (nu)	50 (41 to 58)	37 (29 to 44)*	47 (31 to 64)	52 (40 to 63)^+^
LF/HF	1.4 (0.9 to 1.9)	2.8 (1.6 to 3.9)*	2.1 (0.8 to 3.4)	1.6 (0.1 to 3.1)^+^
Non-linear domain				
SD1	23 (14 to 33)	15 (7 to 24)*	20 (6 to 34)	19 (2 to 36)
SD2	47 (31 to 62)	34 (24 to 43)	32 (19 to 46)	36 (15 to 57)
α1	1.2 (0.6 to 1.8)	1.4 (0.8 to 2.0)	0.95 (0.71 to 1.1)	1.0 (0.8 to 1.1)
α2	1.2 (0.6 to 1.8)	1.2 (0.7 to 1.8)	0.79 (0.66 to 0.91)	1.04 (0.87 to 1.21)
Shannon entropy	3.3 (2.9 to 3.8)	3.4 (3.0 to 3.9)	3.1 (2.8 to 3.3)	3.2 (2.9 to 3.6)
Approximate entropy	1.2 (0.7 to 1.8)	1.2 (0.6 to 1.8)	1.09 (1.0 to 1.1)	1.04 (0.9 to 1.1)
Sample entropy	1.6 (1.1 to 2.2)	1.6 (1.0 to 2.1)	1.5 (1.3 to 1.7)	1.2 (1.1 to 1.4)*

Data are reported as mean and 95% confidence interval (minimum and maximum). *P<0.05, supine *vs* orthostasis within group; ^+^P<0.05, orthostasis *vs* orthostasis between groups (Student's *t-*test). iRR: interval RR standard deviation; HR: heart rate; RMSSD: root mean square differences of successive differences in iRR; RR tri-index: heart rate variability triangular index; LF: power in the low-frequency band; HF: power in high-frequency band; nu: normalized units; α2: alpha 2; α1: alpha 2. Nonlinear analysis provided the plot de Poincaré (SD1 and SD2).

**Figure 3:**
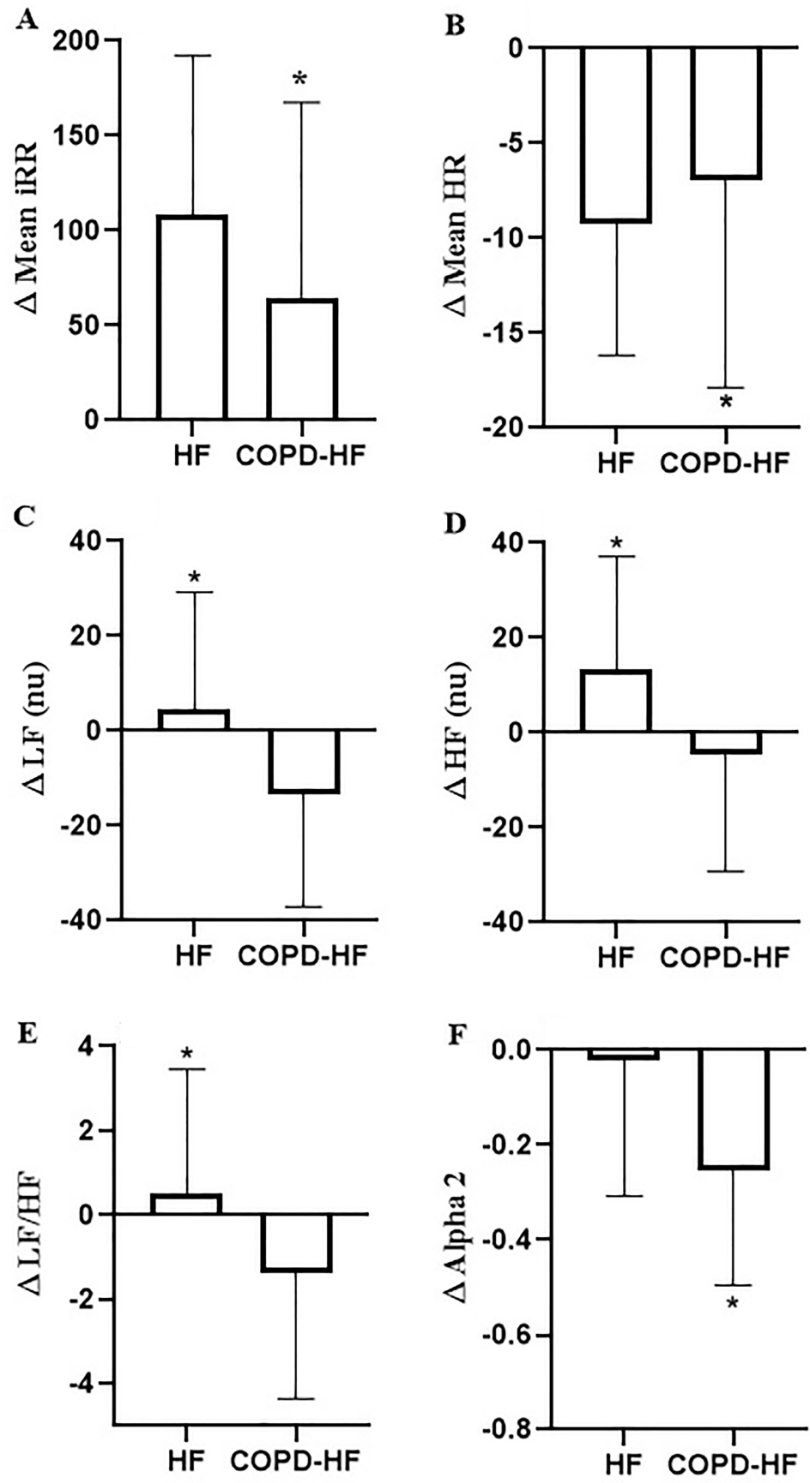
Comparison of heart rate variability indices in Δ active postural maneuver of heart failure (HF) patients and chronic obstructive pulmonary disease-heart failure (COPD-HF) patients. Data are reported as means±SD. *P=0.05 (Student's *t*-test). iRR: R-R intervals; HR: heart rate; LF: low frequency in normalized units; HF nu: high frequency in normalized units; Alpha 2: long-term fluctuations of detrended fluctuation analysis.

In Figure 3, delta (Δ) (Supine-Orthostasis) values showed that COPD-HF patients had a reduction in Δmean iRR, ΔLF, ΔLF/HF, and Δalpha 2, and an increase in Δmean HR and Δ[HF nu] compared with HF patients (P<0.05).

### HRV indices in sitting position and during RSA-M

**Figure 4:**
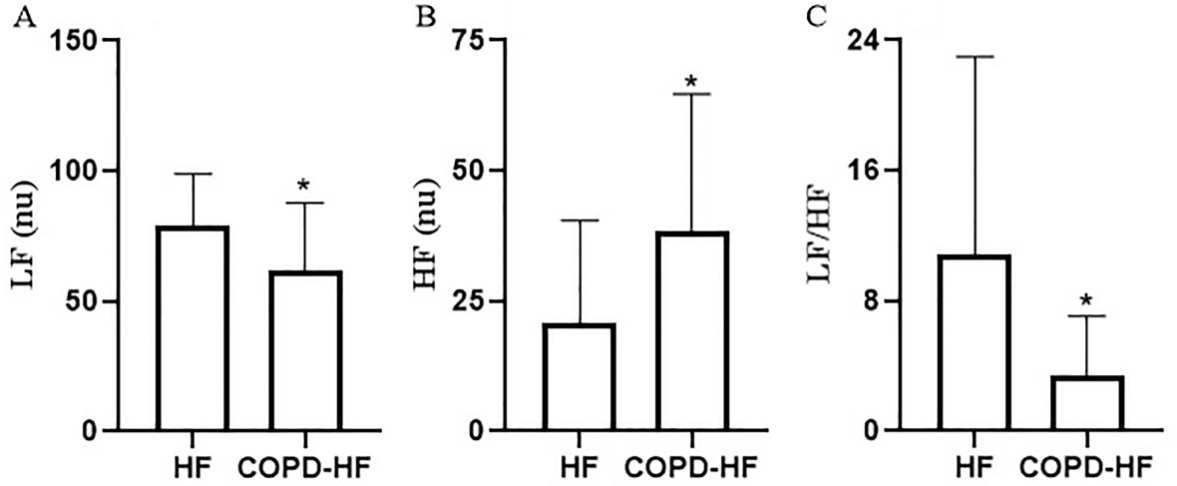
Heart rate variability indices during respiratory sinus arrhythmia maneuver in heart failure (HF) patients and chronic obstructive pulmonary disease-heart failure (COPD-HF) patients. LF: low-frequency band; HF nu: high-frequency band; nu: normalized units; LF/HF: ratio between LF and HF. Data are reported as means±SD. *P<0.05 (Student's *t*-test).

The graph representative of a COPD-HF patient during RSA-M showing lower spectral components compared with an HF patient is shown in Figure 1. We found no significant difference between groups during SB (P>0.05). However, HF patients showed an opposite response during RSA-M, with increased sympathetic modulation (LF nu) and reduced parasympathetic modulation HF nu, (P<0.05) compared with COPD-HF patients (Figure 4).

## Discussion

This is the first study to evaluate the influence of COPD on HF during APM and RSA-M in contrast to HF alone. Our main findings were: i) whereas patients with HF demonstrated, by linear and non-linear indices, positive responses to the APM, only sample entropy was able to demonstrate autonomic responses in COPD-HF patients; and ii) HF patients showed an increased sympathetic modulation and reduced parasympathetic modulation during RSA-M.

### Neural control of heart rate of HF and COPD-HF patients during active postural change

As expected, we found a reduction in mean iRR and HF nu and an increased mean HR and LF nu in HF patients during APM, as blood flow accumulates in the lower limbs, promoted by orthostatic loading, which in turn causes increased sympathetic activity. These findings can be justified by the fact that we excluded patients with functional class IV, that is, those most severely affected and with symptoms at rest, and by the fact that our patients were undergoing optimal clinical treatment. In the present study, all patients were using medications regularly and had mild HF (LVEF 41±5%), as it is known that patients with severe HF may have low HRV associated with vagal reflex loss, resulting in arrhythmic deaths in HF (21).

Roberto et al. (22) observed that HF patients presented sympathetic hyperactivity at rest, which can be attributed to changes in the autonomic system, such as alteration in the sensitivity of peripheral and arterial baroreceptors, increased levels of catecholamine, increased noradrenaline in plasma, increased sympathetic tone, and abnormalities in cardiovascular reflexes (23).

On the other hand, COPD-HF patients did not respond to time and frequency domain indices during APM. Only sample entropy was able to reduce from supine to orthostatic change (P<0.05), demonstrating reduction of HR complexity in that position. Sample entropy is an index able to capture the amount of information contained in a biological signal and characterize a phenomenon complexity and to measure the irregularity of a time series (24,25). This variable, compared to the other nonlinear ones, seems to be more sensitive to postural changes in COPD-HF patients, demonstrating that there is a reduction post-APM in sample entropy, suggesting an increase in sympathetic modulation (24).

When we compared the groups separately in the studied positions, representative indices of frequency domain (nu) showed that patients with the coexistence of COPD-HF presented greater vagal modulation, lower sympathetic modulation, as well as sympatho-vagal balance on orthostatic position compared to HF alone (Table 2, P<0.05). In fact, these results may be explained by the presence of COPD and consequent reduction of FEV_1_, despite of bronchodilators used by this subgroup of patients. In a previous study conducted with only COPD patients, we demonstrated that greater lung function impairment was related to poorer heart rate dynamics during the postural maneuver (26). However, to our knowledge, no study focused on assessing the potential impairment of the HR autonomic response in the coexistence of COPD-HF; therefore, we believe that COPD associated with HF negatively impacts cardiac autonomic modulation during APM.

The effect of COPD on alpha 2 index in HF patients is currently unknown, which is representative of long-term fractal disruption of heart rate when this index is reduced (27). In APM, patients showed a reduction in alpha 2, demonstrating that this maneuver was efficient to show a fractal disruption of HR dynamics.

### Differences of HR neural control between HF and COPD-HF during RSA-M

In the present study, COPD-HF patients presented lower sympathetic response and higher parasympathetic modulation during RSA-M when contrasted with HF alone (see Figure 4). It is already widely known that HF patients present sympathetic hyperactivity in HR control, explained by compensatory changes in the autonomic system caused by disease severity (26–28).

However, in the present study, COPD-HF presented higher parasympathetic activation during RSA-M that could be explained by the oversaturation of this system and thus its inability to increase its response during a purely parasympathetic maneuver. Our findings can be explained by a study by Mazzuco et al. (26) in a subgroup of COPD patients showing depressed responses of parasympathetic modulation during RSA-M, which was attributed to lung hyperinflation.

These results are important since previous study showed that the presence of respiratory symptoms and impaired lung function are predictors of ventricular arrhythmias and cardiovascular mortality (29). In the present study, the COPD-HF coexistence can contribute to potentiate the damage in the autonomous control, producing altered autonomic responses. In this context, controlled breathing techniques, commonly applied during exercise training programs, could contribute to stimulate autonomic nervous control of heart rate, since previous studies on COPD and HF demonstrated that breathing exercises (5,30,31) and physical training (32) may produce benefits to the autonomic nervous system and contribute to reduce morbimortality in these patients (33).

### Clinical implications

Postural change is a common requirement during the day (when waking up, getting out of bed, or getting up from a chair) and needs the integrity of autonomic control so that there are no symptoms of visual turbidity, dizziness, and even falling.

In addition, respiratory maneuvers that involve slow and deep breaths raise parasympathetic tone and consequent mental control, being important techniques to be taught to patients, especially those with chronic cardiorespiratory diseases (9,34). In this context, the new findings presented in this study on the response of COPD-HF patients during APM and RSA-M may lead to an effective improvement in pulmonary rehabilitation in a clinical setting.

### Limitations

Some limitations were present in the study. Although having an effective sample, it was not possible to recruit patients with NYHA and MRC functional grade IV patients, since these patients were excluded from the study for numerous reasons described in the exclusion criteria section. Finally, patients were from only one medical specialty center, and multicenter studies that perform different clinical treatments on patients with COPD-HF coexistence are required.

### Conclusion

COPD directly influenced cardiac autonomic modulation during active postural change and controlled breathing, demonstrating an autonomic imbalance during these maneuvers for patients with COPD-HF coexistence compared with isolated HF. These results reinforced the importance of strategies that could restore cardiac autonomic responses such as respiratory exercises and physical exercise training programs in these patients.
